# CHIME: CMOS-Hosted *in vivo* Microelectrodes for Massively Scalable Neuronal Recordings

**DOI:** 10.3389/fnins.2020.00834

**Published:** 2020-08-11

**Authors:** Mihaly Kollo, Romeo Racz, Mina-Elraheb Hanna, Abdulmalik Obaid, Matthew R. Angle, William Wray, Yifan Kong, Jan Müller, Andreas Hierlemann, Nicholas A. Melosh, Andreas T. Schaefer

**Affiliations:** ^1^Neurophysiology of Behaviour Laboratory, Francis Crick Institute, London, United Kingdom; ^2^Department of Neuroscience, Physiology and Pharmacology, University College London, London, United Kingdom; ^3^Department of Materials Science and Engineering, Stanford University, Stanford, CA, United States; ^4^Paradromics, Inc., Austin, TX, United States; ^5^ETH Zürich, Department of Biosystems Science and Engineering, Basel, Switzerland; ^6^MaxWell Biosystems AG, Zurich, Switzerland

**Keywords:** neurotechniques, electrophysiology, extracellular recording and stimulation, *in vivo*, scalable

## Abstract

Mammalian brains consist of 10s of millions to 100s of billions of neurons operating at millisecond time scales, of which current recording techniques only capture a tiny fraction. Recording techniques capable of sampling neural activity at high spatiotemporal resolution have been difficult to scale. The most intensively studied mammalian neuronal networks, such as the neocortex, show a layered architecture, where the optimal recording technology samples densely over large areas. However, the need for application-specific designs as well as the mismatch between the three-dimensional architecture of the brain and largely two-dimensional microfabrication techniques profoundly limits both neurophysiological research and neural prosthetics. Here, we discuss a novel strategy for scalable neuronal recording by combining bundles of glass-ensheathed microwires with large-scale amplifier arrays derived from high-density CMOS *in vitro* MEA systems or high-speed infrared cameras. High signal-to-noise ratio (<25 μV RMS noise floor, SNR up to 25) is achieved due to the high conductivity of core metals in glass-ensheathed microwires allowing for ultrathin metal cores (down to <1 μm) and negligible stray capacitance. Multi-step electrochemical modification of the tip enables ultra-low access impedance with minimal geometric area, which is largely independent of the core diameter. We show that the microwire size can be reduced to virtually eliminate damage to the blood-brain-barrier upon insertion and we demonstrate that microwire arrays can stably record single-unit activity. Combining microwire bundles and CMOS arrays allows for a highly scalable neuronal recording approach, linking the progress in electrical neuronal recordings to the rapid progress in silicon microfabrication. The modular design of the system allows for custom arrangement of recording sites. Our approach of employing bundles of minimally invasive, highly insulated and functionalized microwires to extend a two-dimensional CMOS architecture into the 3rd dimension can be translated to other CMOS arrays, such as electrical stimulation devices.

## Introduction

There is an increasing interest in understanding large nervous systems, such as the brains of mammals including humans. For these investigations, the main obstacle is the large size of the brain and the extensive population coding employed, which makes it essential to monitor the activity of large distributed neuronal ensembles in most behavioral states (Georgopoulos et al., 1986; Lee et al., 1988; Sanger, 2003; Averbeck et al., 2006; Miura et al., 2012; Gschwend et al., 2016). Brain-machine interfaces (BMI) seek to query and control the state of neuronal networks and hold great promise for neuronal prosthetics. It is clear that for efficient measurement and control, the number of monitored units needs to extend way beyond what has been achieved with currently available methods (Chapin, 2004; Stevenson and Kording, 2011).

Electrical recordings are the state-of the art for BMI and prosthetics, can record at millisecond timescales, and have been used to achieve the largest channel-count recordings capable of time-resolving action potentials (Buzsáki, 2004; Du et al., 2011; Berényi et al., 2014; Shobe et al., 2015; Jun et al., 2017). Despite these advances, the silicon-based approaches for large-scale recordings have several limitations: Silicon probes cover only limited space, and recording sites are generally arranged inflexibly along a vertical/axial direction, while in the most studied brain areas [cortex, hippocampus, olfactory bulb (OB)], sampling in broad horizontal layers is best for studying representations. Device integration challenges have so far prevented insertion at high lateral density <400 μm inter-probe distance. Another limiting factor is that incremental iterations in the design are typically subject to long turnaround times associated with ASIC verification, validation, and fabrication. Finally, while each electrode is small, the size of the overall shank and the resulting tissue damage is posing a great limit to scaling by using multiple silicon probes (Buzsáki, 2004; Kozai et al., 2012; Woolley et al., 2013; Prodanov and Delbeke, 2016).

**FIGURE 1:**
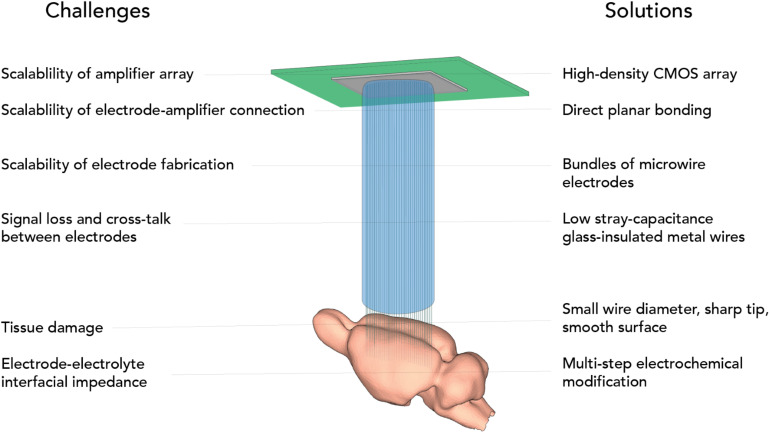
Challenges of scalable electrophysiology addressed by CHIME.

Designing a massively scalable electrophysiological recording system poses challenges at multiple levels and requires a systematic design effort (Figure 1). Apart from the individual electrodes, scaling has been frustrated by the engineering challenge for connectors, amplification, and digitization. One approach to mitigate this has been to integrate electronics, multiplexing before connecting (Lopez et al., 2014; Raducanu et al., 2017; Fiáth et al., 2018), but integrating active electronics requires a largely planar electrode architecture. A further limit to upscaling these approaches is that with further size reduction of individual electrodes, generally coupling impedance and signal loss increase. A potential solution that was suggested is to incorporate active electronics at the recording site (Neves et al., 2008; Lopez et al., 2014; Ruther and Paul, 2015). However, space constraints and temperature limit the scalability of this approach (Marblestone et al., 2013).

All these factors together might underlie the unfortunate situation that scaling has been extremely slow compared to other technologies (Stevenson and Kording, 2011), which speaks to the need for fundamentally new approaches (Marblestone et al., 2013). For slice and culture recordings on the other hand, scaling has advanced more rapidly, by making use of CMOS-based approaches (Scribner et al., 2007; Ferrea et al., 2012; Müller et al., 2015; Dragas et al., 2017; Tsai et al., 2017), however these techniques are inherently only suitable for flat samples, and not for *in vivo* brain recordings in large brains, such as rodents or primates.

Here we present CMOS-Hosted *in vivo* MicroElectrode (CHIME) recordings, an approach to make use of the favorable scaling properties of CMOS devices and extend the flat CMOS architecture into the third dimension using ultra-low-impedance, glass-insulated metal microwire bundles, thus making it suitable for brain recordings (Figure 1). We demonstrate its viability with different CMOS architectures: the Mea1k CMOS MEA chip (Müller et al., 2015) and a repurposed high-speed IR camera circuit, the Cheetah 640CL IR camera ROIC (Neys et al., 2008). We demonstrate connectorization with electrically deposited gold bumps, which is an alternative to the glass-etching based method we presented previously (Obaid et al., 2020), demonstrating the potential to extend CHIME to electrodes of other materials, for example conductive plastics.

## Materials and Methods

Glass-ensheathed gold microwires (ELIRI, S.A., Moldova) were obtained as continuous threads of various lengths (50–500 m) on individual spools. The general process of bundle fabrication is summarized in Supplementary Figure 1. Bundles were created by wrapping microwire from spools with a modified winding machine (Optima 1100, Synthesis, India). The bundle electrodes were then sealed by applying 2-component epoxy resin (Uhu, Germany) on both sides of the bundle. CB 509 polymer (Agar Scientific, United Kingdom) was heated to 126°C in a metal crucible within a hot plate desiccator and then pumped to 0.9 bar for 10 min to remove bubbles from the molten CB. The chip end of each electrode was dipped in molten CB 20 times, removed from the crucible, and hung up for a few minutes to harden. The bundles were centered within a polypropylene tube (2 mL syringe), and the chip end was back-filled with molten CB by pouring from a ladle to fuse it within the tube. The chip end was polished flat by fixing the supporting tube in a custom polishing holder. This holder was designed to fit the pneumatically powered head of a rotating polishing machine (Metaserv 250 with Vector Power Head, Buehler, IL, United States) and could support four electrodes simultaneously. The electrodes were first balanced manually, semi-automatically polished at grit 240 (ANSI), then re-aligned and polished on grit 320 and 600 slurries. The electrode face was then washed with distilled water and inspected with a dissection microscope. The tissue end was embedded in dental cement (Paladur, Kuzler, Germany) by filling the end of another polypropylene tube supported in a mold with freshly mixed liquid. The cement was allowed to harden for 10 min. The cement embedded tissue end was flat polished as described previously.

The embedded polished wires at the tissue end were electrically connected by sputtering gold for 170 s with a sputterer (Agar Scientific, United Kingdom). Next, an insulated macroscopic wire was connected to the sputtered surface with conductive silver epoxy (MG Chemicals, Canada) which was allowed to harden overnight. The silver epoxy was then coated with epoxy resin to insulate and mechanically support the macroscopic wire. Gold bumps were electrodeposited on the chip end to increase the contact surface area of each wire: Briefly, electrodeposition of gold was performed to increase the surface area of the electrical contact on the chip end of bundle electrodes. Hemispherical gold bumps were electrodeposited onto polished bundles from an electrolyte bath containing 10 gL^–1^ KAu(CN)_2_ (Elias et al., 2012). The three-electrode cell setup comprised of a bundle electrode as the working electrode (WE), a platinum rod as the counter electrode, and a Ag| AgCl reference electrode (REF). A potentiostat (Ivium) was used to perform a potentiostatic deposition protocol (−1 V vs REF for 25 s) in a stirred solution heated to 60°C in a chemical hood. After deposition, the bumps were washed with distilled water and visually inspected under a dissection light microscope. Approximately 1 cm of the tissue end was cut off with a hot scalpel blade. The tissue end was embedded in CB but was supported in a polypropylene tube cut at a 30° angle. The tissue end was polished similar to the chip end, but with a custom polishing holder which held electrodes at a 30° angle to result in sharp “needle” tips for simplified tissue insertion. The tissue end was freed from its CB embedding by removing the CB with a solvent stripper (509-S, Agar Scientific, United Kingdom). Removal of CB residue was achieved after immersing in 150 mL of stirred stripper for at least 16 h with the stripper replaced at three different intervals. The freed end was then washed in distilled water. We have presented alternative approach for increasing the metal surface on the chip-end by glass etching in Obaid et al. (2020).

An alternative method was used in a subset of experiments, where both ends of the bundle were embedded into dental cement. After polishing, the tissue end was freed by oxygen plasma ashing (5 h at 100 W, SPI Plasma Prep III).

Gold and iridium oxide (IrOx) were electrodeposited on the tissue end as described in Racz et al. (2019). To electrically connect to the chip end, the embedded gold bumps were pressed into a tightly fitting connector filled with a paste of carbon powder (Sigma) and mineral oil. This created a temporary electrical connection with contact resistance of 100–500 Ω per wire. After electrochemical deposition, the chip end was freed from its CB embedding and the carbon paste was washed off with CB stripper.

The double-functionalized electrode bundle, freed from embedding, was then mounted to a 2 mm × 1 mm × 10 mm flat rod of polystyrene with epoxy resin. Approximately 2–5 mm of the tissue end was left freed on both sides of the bundle electrode. The polystyrene shank was then mounted with 2-part epoxy to a custom-made holder attaching to a custom 3-arm press system (see below). Recordings were performed with either a MEA1k switch-matrix chip (Müller et al., 2015) or a modified Cheetah 640 CL infrared camera (Xenics, Leuven, Belgium). The photosensitive layer of the Cheetah 640 CL was omitted by the manufacturer, so that individual amplifiers at each pixel of the camera chip could be directly accessed via indium bumps. The ∼10 μm pixels have a pitch of 20 μm in a 640 × 512 array. The Cheetah 640 CL can operate in high- and low-gain mode; in the high-gain mode used during recordings, the feedback capacitance is 6.73 fF, sensitivity is 22.8 μV/electron, and the saturating charge is ∼75,000 electrons with 192 electrons of noise (Neys et al., 2008). The reference voltage for the camera (virtual ground of the transimpedance amplifier of every pixel) was approximately 2.3 V. Throughout this work, the camera was operated in integrate-while-read mode, where each frame is integrated while the previous frame is read out through the camera link ports. Depending on the selected window of pixels, recordings took place at frequencies between 1.7 kHz (full frame) and 200 kHz (smallest frame). A DVR System (CORE, IO Industries, Canada) was used to continuously record data at up to 2 GB/s.

For the characterization of the pixel amplifier, an optically isolated, low-noise voltage source was custom-developed (Wray, 2017) to move the pixel within saturation limits by raising the voltage ∼1.65/2.3 V above ground and then applying voltage waveforms with <50 μV noise into a bath containing 0.9% NaCl solution through a standard Ag/AgCl electrode.

Resistance and stray capacitance of glass-ensheathed microwires were measured with an LCR meter (LCR-821, GW-Instek, Taiwan). A three-dimensional printed chamber supported a microwire connected to two ports filled with conductive liquid Galinstan. The resistance across the 7.5 cm wire was measured with both ends of the wire connected to a Galinstan port. An external electrode (copper wire) was immersed along with the connected microwire in PBS solution for various lengths, and the capacitance was measured from the external electrode (in grounded solution surrounding the microwire) to one of the Galinstan ports.

The chip-end of the electrode bundles can be aligned and pressed onto the chip using different solutions: a custom 3-arm press system, built on three micromanipulators (Luigs & Neumann, Germany), joined with a magnetically attached flexible centerpiece and independent strain measurements on each arm (Wray, 2017) was used for recordings with the Cheetah camera chip, and a screw-based passive alignment system (Obaid et al., 2020) with the Mea1k. Pixels contacted to the chip were detected by significant deviations in the average pixel value (mean ± 2 SD).

C57BL/6 mice of both sexes, aged 4–6 weeks, were anesthetized and used for recordings. To expose the OB, craniotomy surgeries were performed as described before (Kollo et al., 2014). For surgery, mice were anesthetized using ketamine (100 mg per kg of body weight) and xylazine (20 mg per kg for induction and 10 mg per kg for maintenance) administered intraperitoneally and supplemented as required. Body temperature was kept at 37°C with a feedback-regulated heating pad (FHC, ME, United States). A custom-made steel headplate was fixed to the parietal and interparietal bone plates with dental cement. A 2 mm × 3 mm craniotomy was drilled over the OB. The chamber surrounding the craniotomy was filled with Ringer’s solution to prevent it from drying out. Next, a Ag/AgCl reference electrode was placed in the meniscus of Ringer’s solution.

After surgery, the anesthetized mouse was brought directly to the recording setup and kept under anesthesia with intraperitoneal supplementation of ketamine/xylazine as needed. The mouse was placed on a platform mounted on a three-axis manipulator below the recording device with the electrode bundle facing downward. Respiration was monitored by a piezoelectric sensor and body temperature maintained at 37°C. Using a small web camera (Raspberry Pi, Raspberry Pi Foundation, United Kingdom) and mirror, the bundle electrode was aligned over the craniotomy. The reference electrode in the meniscus over the brain was lifted to the reference voltage of the camera using a low-noise voltage source (see above). Wire connectivity and noise were coarsely assessed in the meniscus before insertion.

For CT/MRI images, after performing a craniotomy as described above, a bundle was inserted into the brain with a micromanipulator (SM7, Luigs and Neumann). The mouse was then given an overdose of the anesthetic, subsequently decapitated, and the whole head was embedded into a block of epoxy resin (Uhu, Germany) and attached to the microscope specimen holder. The whole head was imaged with a microCT (XRadia 510 Versa, Zeiss, Germany) at 30 kV (50.5 μm pixels, 40-min scan), and, then, the region of interest at the tissue end of the inserted bundle was imaged at higher resolution (3.98 μm pixels, 10-h scan). Imaris software was used to extract and semi-manually delineate the features (bundle, skull and jaw bone, and the soft tissue). All animal experiments were approved by the local ethics panel and the United Kingdom Home Office under the Animals (Scientific Procedures) Act 1986.

## Results

### Microwire Bundle Fabrication

**FIGURE 2:**
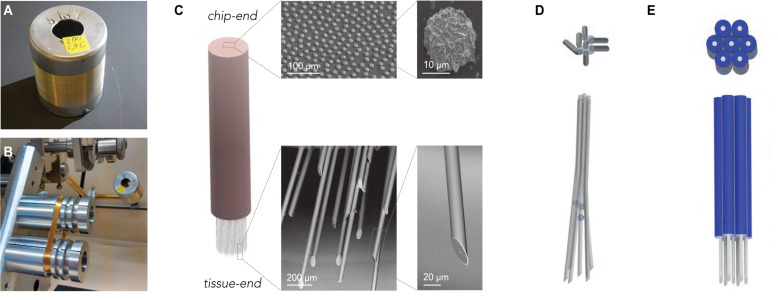
Scalable fabrication of CHIME electrode bundles. **(A)** Cast glass-insulated metal microwires are produced in several-km-long, continuously conductive stretches. **(B)** The flexibility and robustness of the composite wires allow the preparation of large wire-count bundles by spooling. The image shows a 1000-wire bundle prepared on a fiber winding machine. **(C)** The bundle is embedded into polymer, and both ends of the fiber bundle are altered with scalable procedures. For optimal contact with the electronics, the chip-end of the embedded bundle is polished flat, and the exposed metal conductor cores at the chip-end of the wires are increased by electrochemical gold deposition (SEM images top row). The tissue-end of the wires is sharpened to limit tissue damage (SEM images bottom row) and electrochemically modified to optimize recording capabilities in the extracellular electrolyte (not shown, see Racz et al., 2019). **(D)** Regular electrode spacing achieved by a sacrificial layer deposited onto the microwires before bundling.

An ideal electrode for scalable neuronal recordings has good signal-to-noise characteristics, penetrates into the tissue without causing significant damage, while a large number of channels can be realized without excessive fabrication efforts. Volume displacement, and friction can impact on the tissue-electrode interaction upon insertion (Kozai et al., 2010, 2012). Therefore, we conclude that ideal microwires should be small, sharp and smooth. At the same time, to achieve large signal-to-noise ratio, recordings should be focal, where the geometric electrode area is below a few μm^2^ (Teleñczuk et al., 2018). To achieve all this, we used a modified Taylor–Ulitovsky method (Zhukov, 2006; Baranov et al., 2017) that allows for fabrication of kilometer-long, glass insulated microwires with conductive metal cores (Figure 2A).

The thermal drawing process used for producing the wires allows for great flexibility in the dimensions and the inner/outer diameter (ID/OD) ratio. Wire geometry can be adjusted by varying parameters, such as heating and pulling speed, and dimensions as small as 10 μm can be achieved, with nanoscale conductive cores (Ioisher et al., 2011; Yaman et al., 2011). The outer glass surface of the electrodes is highly consistent and smooth. The microscale composite wires are mechanically flexible, allowing wrapping as an approach to bundle electrodes. This ensures easy scalability (Badinter et al., 2010) up to hundreds of thousands to millions of channels (Figure 2B). In the recordings presented here, electrodes with 22–25 μm OD and 1–7 μm ID were used.

Recording arrays from the microwires were prepared from spools to make bundles of 100–1000 wires (Supplementary Figure 1). First, we embedded the wire bundle into hard plastic for polishing. The chip-end was embedded permanently until the end of the process, while the tissue end was only temporarily embedded and later freed, for which we either used a solvent-removable hard plastic (CB 509) or also PMMA for subsequent plasma ashing (see below). The chip-end and the tissue-end of the bundle were finely polished with a grinder polisher. Larger metal surfaces provide better contact interfaces, but wire diameter is a critical factor for tissue damage. To uncouple these two factors and achieve optimal connection to the CMOS array, after polishing, we increased the exposed metal cores of the microwires by electrochemical metal deposition (Figure 2C). Before spooling, to ensure that local tissue displacement stays limited across the electrode array, the bundles were, in some experiments, spaced by incorporating a sacrificial spacer material during bundle fabrication (Figure 2D; Obaid et al., 2020).

The tissue-end was angle-polished at 30° to achieve sharp wire tips, and the embedding material was removed either by oxygen plasma (for PMMA) or organic solvents (for CB 509), to free the last 2 mm of the tissue-end of the microwires (see section “Materials and Methods” for details and alternatives).

### Electronics

While wires can be easily made in kilometer length and both electrochemical modification and polishing is inherently scalable and highly reproducible due to the standardized surface, using individual conventional amplifiers and operating amplifiers in general is not. CMOS integrated circuits on the other hand provide extensive scalability.

**FIGURE 3:**
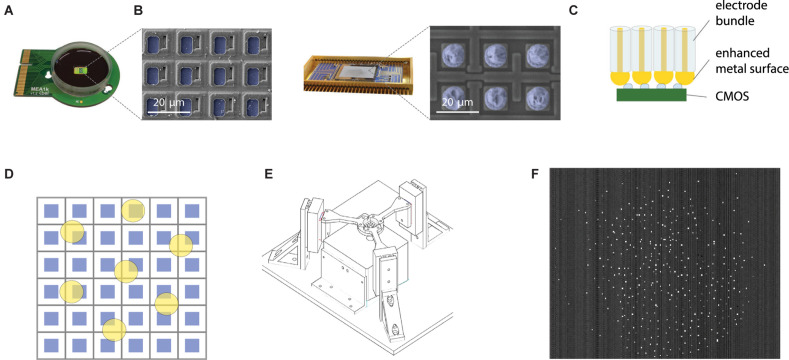
Scalable direct bonding of electrodes to CMOS pixels. **(A)** Mea1k, a high-density, high signal-to-noise, switch-matrix based MEA chip (Müller et al., 2015). Inset: SEM of the chip surface. Blue pseudo-colored areas denote the active Pt recording sites. **(B)** Commercial IR sensors provide another class of high pixel-count CMOS amplifier array, capable of sampling from >10^5^ pixels at rates of several kHz (here: Xenics Cheetah 640CL, Neys et al., 2008). Blue pseudocolor in the SEM image indicates Indium bumps on the amplifier input sites. **(C)** Direct contacting of electrode bundles to the conductive pixels is a highly scalable strategy to electrically interface bundles to electrode arrays. Increasing the size of the contacting metal surface of the microwires can be achieved in multiple ways and can enhance contact quality (e.g., Figure 2C). **(D)** High yield of contacts can be achieved without the alignment of electrode cores (yellow) and pixel recording areas (blue) by using sufficient spacing and large diameter electrode cores. **(E)** Alignment of the recording bundle and the flat CMOS array was achieved by a three-arm force-sensitive micromanipulator system. **(F)** A wire bundle of 200 electrodes connected to the amplifier array of an IR CMOS sensor. Bright pixels indicate connected electrodes, due to a small offset between the animal and the reference of the pixel amplifier.

CMOS based high-density MEA chips are specifically designed for recording extracellular signals from brain tissue (Figure 3A). In addition to the high signal-to-noise ratio, further advantages are the capability for electrical stimulation and subselection of active pixels (Jones et al., 2011; Ferrea et al., 2012; Müller et al., 2015). Even higher channel-count CMOS amplifier arrays are available in commercial IR sensors tailored to amplify small signals at high frame rate. In fact, they were shown to be, in principle, useable for slice MEA recordings (Scribner et al., 2007). We thus characterized an IR sensor, the Xenics Cheetah 640CL for its suitability to be used as a scalable amplifier platform for *in vivo* neural recordings. This IR camera is capable of frame rates of up to 200 kHz (1.7 kHz full frame), which allows for a sufficiently high sampling rate to resolve neuronal action potentials. The CMOS readout circuit contains 327,680 capacitive transimpedance amplifiers with small feedback capacitances (7 fF, Neys et al., 2008). In the IR camera, the readout circuit is connected to photodiode layer using Indium bump bonding (Vermeiren et al., 2009). Individual amplifiers can thus be accessed through the Indium bumps (Figure 3B).

### Connecting Bundle and Chip

Contacting the microwire bundle to the CMOS chip in a scalable manner was achieved by a direct contacting approach (Figure 3C). Exploiting the high pixel count and density of the CMOS recording arrays, high-yield contacts were established by spacing the wires sufficiently (>100 μm, see Obaid et al., 2020), and concurrently increasing the contacting area of individual wires (Figure 3D). With this approach, each wire is connected to a pixel with high probability, while single CMOS pixels are never connected to two wires. The chip end of the bundle was embedded into PMMA, and finely polished. To provide a sufficiently large contact surface for each electrode, we electrochemically deposited 10 μm gold bumps onto the chip-end of the wires. The bundle was pressed against the ROIC (read-out circuit) using a custom designed press (Figure 3E) and held in place using the press during the whole duration of the experiment. After removal of the bundle from the chip, the gold bumps showed deformation and indentations made by the CMOS surface, suggesting strong and stable contact. Connected pixels are sparsely distributed over the chip area (Figure 3F).

### Signal-to-Noise

**FIGURE 4:**
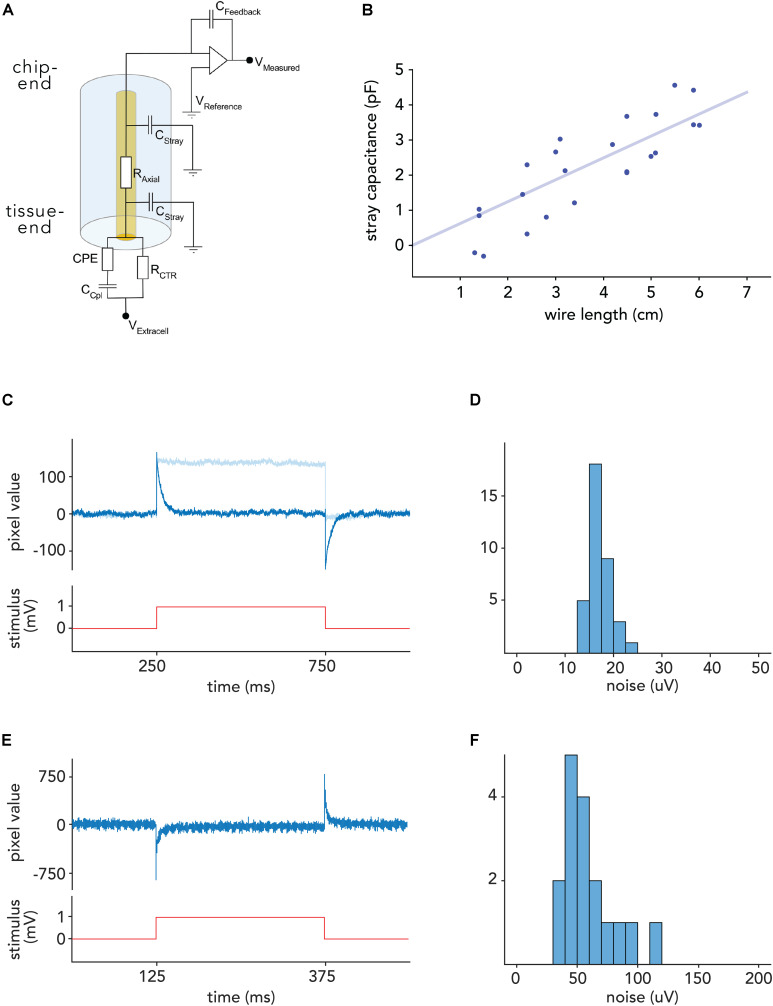
Noise levels in voltage recordings with CHIME. **(A)** Electrical model of the recording configuration [depicted for a capacitive transimpedance amplifier derived from IR sensors (Vermeiren et al., 2009)]. The axial resistance of the metal core microwires (R_Axial_) is low (<300 Ω/cm). Signal loss is mainly determined by the stray capacitance (C_Stray_). Signal amplitudes depend on the feedback capacitance of the amplifier (C_Feedback_), and the degree of interfacial impedance, primarily the coupling capacitance (C_Cpl_). **(B)** Stray capacitance for a glass/gold wire with outer diameter of 28.3 μm and inner diameter of 1.1 μm. Stray capacitance scales linearly with wire length and is low (<1 pF/cm) due to the high outer to inner diameter ratio. **(C)** Response of one pixel of a Mea1k switch-matrix based MEA (Müller et al., 2015) to a 1 mV voltage step applied to the bath, recorded through a connected electrode. On-chip high-pass filter: 300 Hz (dark blue), 1 Hz (light blue) **(D)** RMS noise level distribution in connected Mea1k pixels. **(E)** Voltage response of a Cheetah 640CL IR camera ROIC pixel in arbitrary units (Neys et al., 2008). Due to the inverting amplifier circuit, responses are reversed. **(F)** Noise level distribution in connected IR camera CMOS pixels.

Electrical signals in the extracellular space originating from single cells have typically amplitudes of few tens to hundreds of microvolts in close vicinity to the cell soma and rapidly decay with distance. The signal amplitude, picked up by any electrophysiological recording device, is limited by the interfacial impedance between the solid-state conductor and the extracellular electrolyte (Figure 4A) and the properties of the amplifier. Signal loss and baseline noise levels in deep brain recordings are in most cases limited by stray capacitance. The stray capacitance depends on the insulating material and the ratio of the OD and ID. Thus, glass/metal microwires with ODs of 10–20 μm and an ID of the metal core as small as 1 μm are theoretically predicted to have minimal stray capacitance due to the large OD/ID ratio provided that pulled glass features the same electrical properties as bulk glass. To assess this, we immersed distinct lengths of wire into electrolyte, and measured the stray capacitance directly. The measurements confirmed a very low stray capacitance for the glass/metal composite wires (less than 80 fF/mm, Figure 4B). While the small core and resulting large OD/ID ratio ensures minimal stray capacitance, the small exposed metal electrode surface at the tip of the electrode, in principle, could entail a large electrode impedance. To address this issue, we designed an electrodeposition protocol that lowers the specific impedance of the electrode-electrolyte interface and can be performed on an arbitrary number of electrodes simultaneously (see Racz et al., 2019).

To probe the ability of CMOS pixel amplifiers to record small voltage fluctuations expected during recordings, we connected bundles, electrochemically modified as described above, to two types of CMOS chips (MEA1k: Müller et al., 2015, and Xenics Cheetah 640-CL), and submerged the tissue-end into a voltage-clamped bath.

In order to compensate for the reference voltage offset of the chips (1.65 V for the MEA1k and 2.3 V for the Cheetah 640-CL, Neys et al., 2008), the bath was voltage-clamped using custom electronics to the appropriate offset (see section “Materials and Methods”). For the Cheetah CMOS system, pixels were activated by large (2–3 V) voltage steps to break any potential residual oxide layer (Gemme et al., 2001). We characterized the electronics by measuring the response to 1 mV square and sinusoidal voltage signals, applied to a bath of saline (Figures 4C–F), and found that individual pixels are capable of recording voltage signals with a noise floor of 24.2 ± 7.7 μV (RMS) on the MEA1k chip, and 58.2 ± 21.5 μV for the Cheetah 640-CL. This resulted in signal-to-noise ratios of 6–20 and 2.5–9, respectively, given 150–500 μV spike amplitudes, which were recorded reliably in the mouse brain. After subtracting correlated noise, the residual RMS noise was 6.5 ± 2.6 μV in connected MEA1k pixels, suggesting that noise levels could be further reduced by shielding.

### Bundle Design

The fundamental limit to scalability of electrodes is often considered to be tissue damage (Marblestone et al., 2013). The size of the implant is an important factor, and it has been suggested that small wires <20 μm would not cause significant damage to the brain tissue (Kozai et al., 2012; Guitchounts et al., 2013). Extensive surface tension due to penetration force, increased pressure due to volume displacement and a potential direct effect of friction of the moving electrode are thought to be the underlying reasons for tissue injury (Edell et al., 1992; Subbaroyan et al., 2005). Beyond the direct damage to neurons, the rupture of vessels is a strong indicator of tissue damage, and a predictor of subsequent degradation of recordings (Kozai et al., 2012; Prodanov and Delbeke, 2016). Histology after insertion experiments with single CHIME electrodes indicated minimal damage to the vasculature (see Racz et al., 2019 and Obaid et al., 2020 for detailed discussion of vascular damage). In case of large channel-count electrode bundles, a high density of recording sites is preferred. For bundle implantations, there is likely a trade-off between the density of recording sites and amount of damage caused, making the spacing between shanks an important factor. Considering these factors, we tested bundles of 200–500 wires with an average inter-wire spacing of around 100 μm.

**FIGURE 5:**
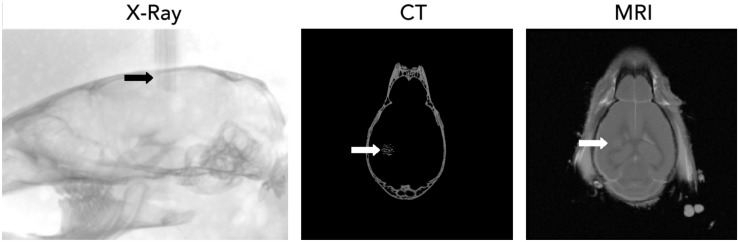
Localization of CHIME microwire bundles using X-ray computer tomography (CT, left: individual X-ray projection) and magnetic resonance imaging (MRI).

The tissue-end of the wires were sharpened and prepared as described above and inserted into the cortical tissue after the removal of the dura mater. Wires inserted readily into the tissue, without buckling, as expected from estimation of the buckling force for the glass-metal microwires (≈300 μN for a mm long microwire). For the localization of recording sites, the electrode bundle was visualized inside the skull (Figure 5) by three-dimensional X-ray computed tomography. Individual microwires could be visualized. Apart from stereotaxic coordinates relative to bone landmarks, further delineation of the anatomical location of each recording site is possible with correlated MRI (Figure 5).

### Neuronal Recordings With CHIME

**FIGURE 6:**
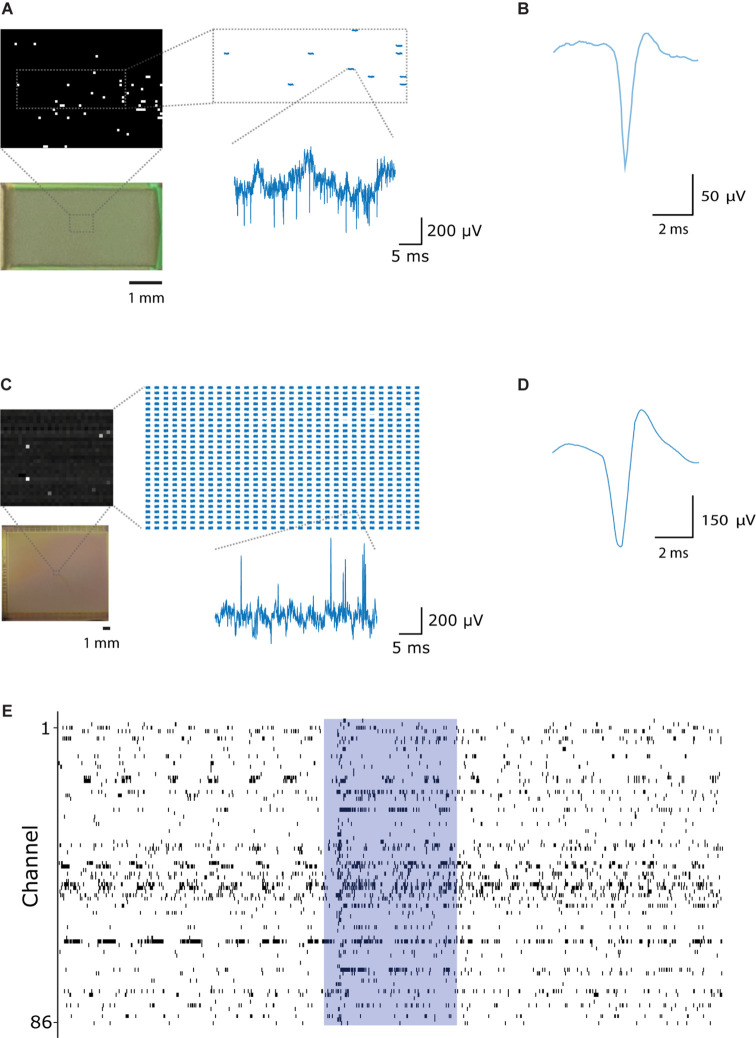
Large-scale, parallel electrophysiological recordings with CHIME. **(A)** Switch-matrix based MEA chips allow for recording selectively from the pixels connected to electrodes. The inset shows traces recorded within the frame. Only pixels connected to electrodes are pre-selected and recorded. Voltage trace scale bars: 5 ms and 200 μV. **(B)** Average spike waveform recorded from the mouse main olfactory bulb with the switch-matrix MEA chip (Müller et al., 2015). **(C)** CMOS IR camera chips (Neys et al., 2008) provide a frame stream from a large number of pixels. All pixels are recorded in a pre-selected rectangular region, of which only a subset is connected to electrodes. Scale bars: 5 ms and 200 μV. **(D)** Average spike waveform recorded from the mouse main olfactory bulb with the IR camera chip. **(E)** Spike events recorded from 86 channels in the mouse main olfactory bulb with the Mea1k CMOS. The blue area indicates the timing of a 1 s long odor stimulus.

Finally, we performed *in vivo* neuronal recordings using the here described CHIME assembly with both chips (Figure 6). The switch-matrix based CMOS MEA architecture (Müller et al., 2015) has the advantage that recorded channels can be pre-selected (1024 channels from 26,400), thus it is not necessary to stream and record pixels not connected to recording electrodes. Additionally, programmable high-pass filter, DC-offset cancelation and voltage or current stimulation through individual pixels/electrodes is available. In comparison, a ROIC repurposed from a CMOS IR camera (Xenics Cheetah 640-CL) provides 327,680 simultaneously useable pixel amplifiers. As offset compensation or filtering is not possible in this architecture, large drifts more often lead to pixel saturation. In case of the IR CMOS camera, voltage traces can be extracted from connected pixels *post hoc* from the video stream recorded by a DVR system.

We recorded neuronal activity from the mouse OB with CHIME using both CMOS systems. With the MEA1k, for bundles of 200 wires, 156 ± 36 pixels were found to be connected with good signal-to-noise ratio (*n* = 7 experiments with the Mea1k CMOS). Recordings started immediately after implantation. Recording sessions lasted 23 min to 2 h, with an average total duration of 63 min, measuring at different locations for 10-40 min. Connected electrodes recorded large-amplitude LFP signals (Figure 6A), and high signal-to-noise single-unit spikes (Figure 6B and Supplementary Figure 2). The IR-camera chip provided lower amplitude LFP signals, due to the high-pass filtering characteristic of the pixel amplifier (Figure 6C), but well isolated single unit spikes could be recorded on individual channels (Figure 6D).

The CHIME technique provides a means for scalable, high-temporal resolution, parallel recordings from mammalian brain circuits at high lateral density (Figure 6E).

## Discussion

Here we have used flexible, glass insulated, metal microwires, connected to a CMOS readout circuit, to perform electrical recordings from neurons. The described technique, CHIME recordings, promises to drastically increase the number of simultaneously recordable neurons. Recording sites can be arranged in arbitrary locations, allowing the experimenter to query neuronal networks *in vivo*, such that the readout arrangement is driven by the addressed functional question rather than the physical limitations of the recording system.

What are the limits to the scalability? At the level of the amplifier array, the number of channels in CMOS chips increases exponentially (Moore, 2006), and the latest designs already substantially surpassed the ≈300,000 channels available in the Xenics Cheetah 640CL camera. Imaging chips have the potential to continue to scale following Moore’s law, reaching 10^7^–10^8^ channels in the near future. MEA chips, which are routinely used to record from cultured cells, brain slices or flat preparations, such as the retina, also show rapid development, and chips with significantly larger pixel counts are under development (e.g. Sahasrabuddhe et al., 2020). While the connectivity between electrodes and chip did pose development challenges, we have not seen any decrease in the efficiency of connection in larger bundles (Obaid et al., 2020). At the scale of the electrode bundles, achieving a sufficiently flat surface for reliable connection is not too demanding and far from the standards needed for, e.g., silicon wafers. Due to the flexibility of the wires, the fabrication of electrode bundles is inherently scalable. A potential limit to channel counts could result from tissue damage.

The development of modern silicon probes has resulted in reduced local damage upon insertion and, in many cases, allows for recording briefly after insertion. However, depending on brain area and insertion parameters, most experimenters experience significant improvements in signal-to-noise ratios in the first 40–60 min (c.f. Fiáth et al., 2018). This is generally attributed to local tissue disturbance, and acute damage to the blood-brain-barrier (Bjornsson et al., 2006; Kozai et al., 2012). While with adjustments of insertion speed, the initial dip in S/N can be minimized (Fiáth et al., 2018), there is trade-off between more local damage during fast insertions, and increased tissue dragging and tension using medium-slow insertion speed.

Despite the fact that CHIME electrodes were inserted at high speed (≥100 μm/s), they provided stable signals from the beginning of the recordings (Supplementary Figure 3). Wires of 15–20 μm diameter cause minimal damage to the blood-brain-barrier. This can be demonstrated using Evans Blue dye injected into the bloodstream, followed by the insertion of the electrodes into the brain, and perfusion fixation. As Evans Blue is bound to albumin, its presence in the brain tissue would indicate damage to the blood-brain-barrier (Rawson, 1943; Belayev et al., 1996). Such experiments confirmed minimal damage upon insertion of sharp 20 μm glass/metal fibers with an average spacing of 100 μm, markedly less compared to the extravasation caused by a standard silicon probe (Racz et al., 2019; Obaid et al., 2020), supporting the notion that damage to the tissue depends non-linearly on the size of the electrode. We also have seen that blood vessels are pushed away by the electrode tip during penetration (Racz et al., 2019), which prevents bleeding and subsequent degradation.

The small size of wires and smooth surface also provides mechanical compliance (Seymour and Kipke, 2007; Etemadi et al., 2016; Luan et al., 2017) and thus likely enhanced biocompatibility. With increasing number of wires, we expect at some point tissue displacement to become the limiting factor. As a solution, wires could be made smaller. With smaller wire diameters, however, at some point the tip impedance becomes too high, impeding signal strength. As a solution, novel materials could be used on the electrode-electrolyte interface, which further increase the specific surface area and improve coupling (Kim et al., 2010; Prasad and Sanchez, 2012; Baneyx and Park, 2013; Chung et al., 2015). Considering the input impedance of the amplifiers, when using 15–20 μm wires, tip impedance is magnitudes lower than what would become a limiting factor, and further improvements can be made for smaller probes.

With small wires, another challenge is posed by buckling. The critical buckling force decreases with the fourth power of the radius, therefore with decreasing size, the force that the wire can exert onto the tip drops significantly. This causes problems if the maximal force is below the force needed for insertion. Measurements have been made for different types of electrodes (Sharp et al., 2009; Casanova et al., 2014; Fekete et al., 2015), and recently a quantitative study was undertaken to understand the penetration mechanics of microwires specifically (Obaid et al., 2018). In our experiments, 15–20-μm-diameter wires could be inserted into the cortex several millimeters deep without support. For thinner wires, a possible solution is the use of temporary coating agents (Foley et al., 2009; Lewitus et al., 2011; Etemadi et al., 2016; Singh et al., 2016). Wire fabrication with the Taylor–Ulitovsky method allows for direct fabrication of approximately 10-μm-diameter wires. Smaller dimensions can be achieved by using a three-component pre-form, with a conductive core, glass inner cladding and etchable glass outer cladding. The etchable glass could then be specifically removed by acids during bundle fabrication analogously to the fabrication of nanochannel array glass (Tonucci et al., 1992) or leached fiber bundles (Gerstner et al., 2004). Another approach is to use thermal drawing to produce tapering in the electrodes (Ioisher et al., 2011; Yaman et al., 2011).

Flexible wires do not necessarily follow a perfectly straight trajectory through the tissue; while this feature allows them to, e.g., avoid blood vessels, it renders positioning more difficult compared to larger or rigid probes, and the random arrangement of recording sites means that devising reliable spike sorting algorithms is more challenging, compared, e.g., to silicon probes with fixed, regularly spaced recording sites. Approximate electrode locations can be adjusted by the spacing of wires during bundle fabrication, however the recording sites are not necessarily homogeneously distributed. Nevertheless, when the number and average density of recordings sites allows the recording of virtually all units through multiple channels, this arrangement has less importance. Direct localization of the wires can be achieved by high-resolution X-ray tomography (Figure 5), combined with other methods to identify specific brain areas and layers. Another possible approaches to localize recording sites include the use of “electrical imaging,” whereby the analysis of noise and signal correlations between channels can be exploited to predict their relative location (Cybulski et al., 2015) and inter-wire impedance measurements.

While glass wires provide many advantages including high insulation and smoothness, other types of wires could be used as they get optimized and become available in large quantities. Carbon fibers (Guitchounts et al., 2013) have the advantage of stiffness, and good recording properties, while fabrication processes are, as of now, less scalable, and the fibers have a higher stray capacitance and less flexibility. Plastic wires can also be used (Canales et al., 2015) and have the advantage of great flexibility, and tissue compatibility. On the other hand, the metals that can be used at the low temperatures currently needed for plastic fiber drawing (e.g., tin), provide a less advantageous substrate for electrochemical modifications compared to higher melting-point metals, such as gold or copper, and might require larger core diameters (and thus increased stray loss) due to reduced electrical conductivity.

As with other large-scale approaches, data handling and, in particular, data analysis will be a key challenge. Identifying and isolating spiking activity from individual neurons is an important requirement and is typically resolved by clustering based on waveforms. Large amplitude spikes are both more informative on the waveform and originate from a smaller volume around the electrode. Therefore, the extent of local damage around the electrode has a strong impact on the reliability of the assignment of spikes to individual neurons, which points to the advantage of small microwires.

The system described here is currently designed for head-fixed experiments. However, the extremely low stray capacitance (<100 fF/mm) makes it possible to record through long (10 s of cm) wire bundles, thus immediately allowing tethering. In addition, the CMOS chip itself is relatively small, it is thus furthermore conceivable – at least in larger mammals – to abandon tethering altogether, move the active electronics to a location outside the skull and perform signal processing, compression, and ultimately wireless signal transmission there. We found that amplitudes and waveforms remained stable for over 40 min (Supplementary Figure 3) with CHIME, but for chronic recordings, lasting days to months, assessment of chronic damage with further histological and functional studies will be necessary.

## Conclusion

In summary, combining microwire bundles and integrated circuit arrays allows for a highly scalable neuronal recording approach, essentially tying the progress of electrical neuronal recording to the rapid progress in silicon microfabrication. Moreover, our approach of employing bundles of minimally invasive, highly insulated and functionalized microwires as a means to extend a two-dimensional CMOS architecture into the 3rd dimension can be translated to other integrated circuit arrays, such as electrical stimulation devices, and, thus, might provide a pathway for high-bandwidth bidirectional interfacing with neural circuitry.

## Data Availability Statement

The datasets generated for this study are available on request to the corresponding author.

## Ethics Statement

All animal experiments were approved by the Ethics Committee of the Board of The Francis Crick Institute and the United Kingdom Home Office under the Animals (Scientific Procedures) Act 1986.

## Author Contributions

MK, RR, ME-H, AO, MA, AS, and NM initiated the concept and design. MK, RR, ME-H, AO, YK, and WW developed the techniques required for the experiments. MK, RR, and WW performed the experiments, data acquisition, and data analysis. AH and JM developed CMOS recording electronics used in the experiments. MK and AS wrote the manuscript with support from RR, NM, ME-H, JM, and AH. All authors contributed to the article and approved the submitted version.

## Conflict of Interest

AS, MA, NM, YK, and ME-H are employed by and/or hold shares in Paradromics, Inc., a brain-computer interface company. JM is a co-founder of MaxWell Biosystems AG. The remaining authors declare that the research was conducted in the absence of any commercial or financial relationships that could be construed as a potential conflict of interest.
