# Supplementary material for: CHIME: CMOS-Hosted in vivo Microelectrodes for Massively Scalable Neuronal Recordings
Source: Front Neurosci. 2020 Aug 11;14:834. doi: 10.3389/fnins.2020.00834 (PMC7432274; doi:10.3389/fnins.2020.00834)
Supplement: Supplementary file 1 [file Image_1.pdf]

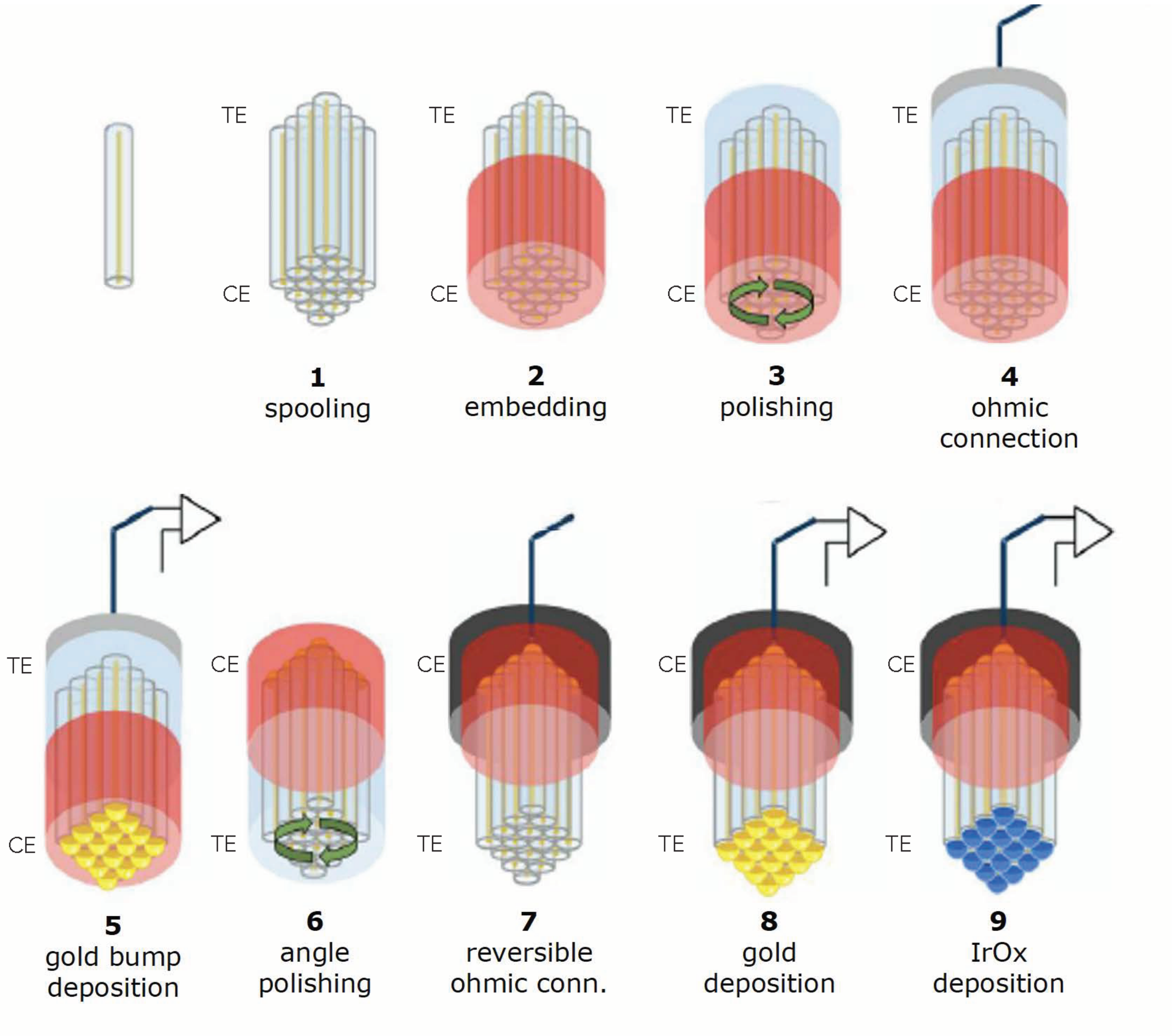

Supplementary Figure 1. The CHIME bundle fabrication process.

In the first phase (**1-5**), the connecting surface of the chip-end (CE) of the bundle is prepared. First, spaced or unspaced wires were bundled (**1**), and embedded into hard plastic (**2**, *pink*) for polishing (**3**). The tissue-end (TE) of the bundle is embedded into a sacrificial plastic embedding material (transparent blue, different materials can be used, see Materials and Methods), which can be later removed, and also polished. After polishing the CE, an ohmic connection is established to the wire cores through a layer of sputtered gold and silver-epoxy (**4**). On the core of each wire on the CE, a gold bump is grown by electrodeposition (**5**). Following this, the TE is polished at an angle to achieve sharp probe tips (**6**), and the TE sacrificial embedding material is removed to free the electrode bundle. Through the gold bumps, a transient electrical connection is established using a carbon-based connective paste (**7**). Finally, the electrodes are functionalized in a two-step electrodeposition process (**8, 9**; cf. Racz et al., 2019), the temporary carbon-paste connection is removed, and the bundle is washed.

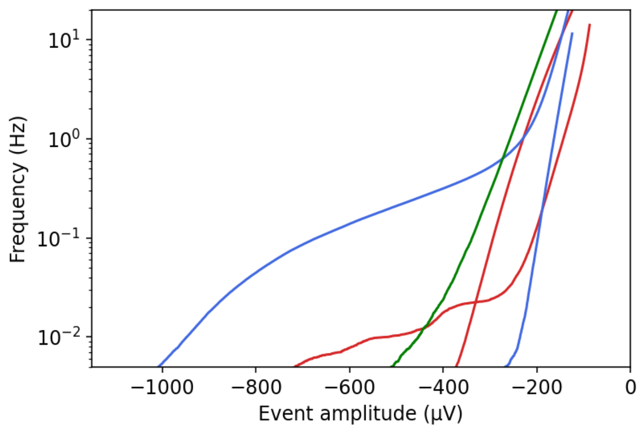

Supplementary Figure 2. Distribution of event amplitudes.
Frequencies of spike events with negative peaks (>4*SD) per channel are given across all channels in 5 experiments.
(Recording area: red - main olfactory bulb, blue - neocortex, green - pirifom cortex).

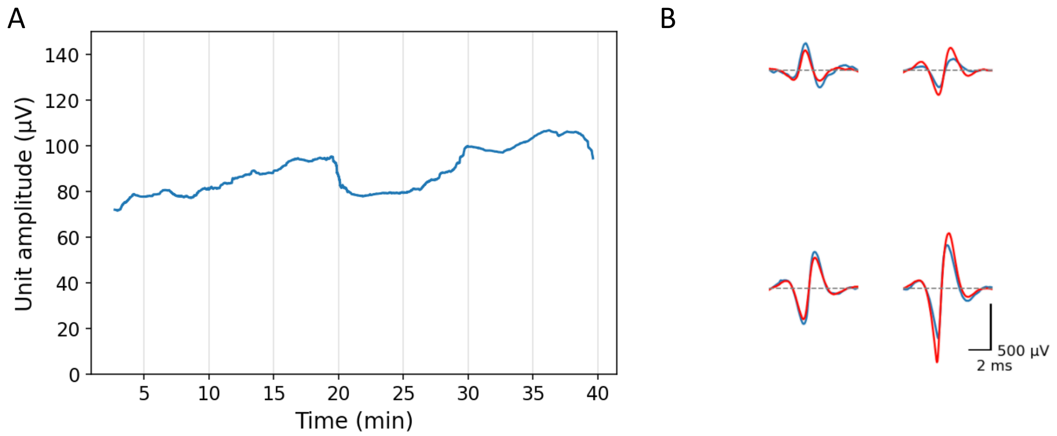

Supplementary Figure 3. Recording stability.
A) Change of mean amplitude of sorted units during a CHIME recording (moving average, τ=5 min). **B)** Average spike waveforms of sorted units during the first half (blue) and second half (red) of the recording.
